# Supplementary material for: Forward models demonstrate that repetition suppression is best modelled by local neural scaling
Source: Nat Commun. 2018 Sep 21;9:3854. doi: 10.1038/s41467-018-05957-0 (PMC6154964; doi:10.1038/s41467-018-05957-0)
Supplement: Supplementary file 1 — Supplementary Information [file 41467_2018_5957_MOESM1_ESM.pdf]

Supplementary Material

From neurons to voxels - repetition suppression is best modelled by local neural scaling

Alink et al., Nature Communication (2018)

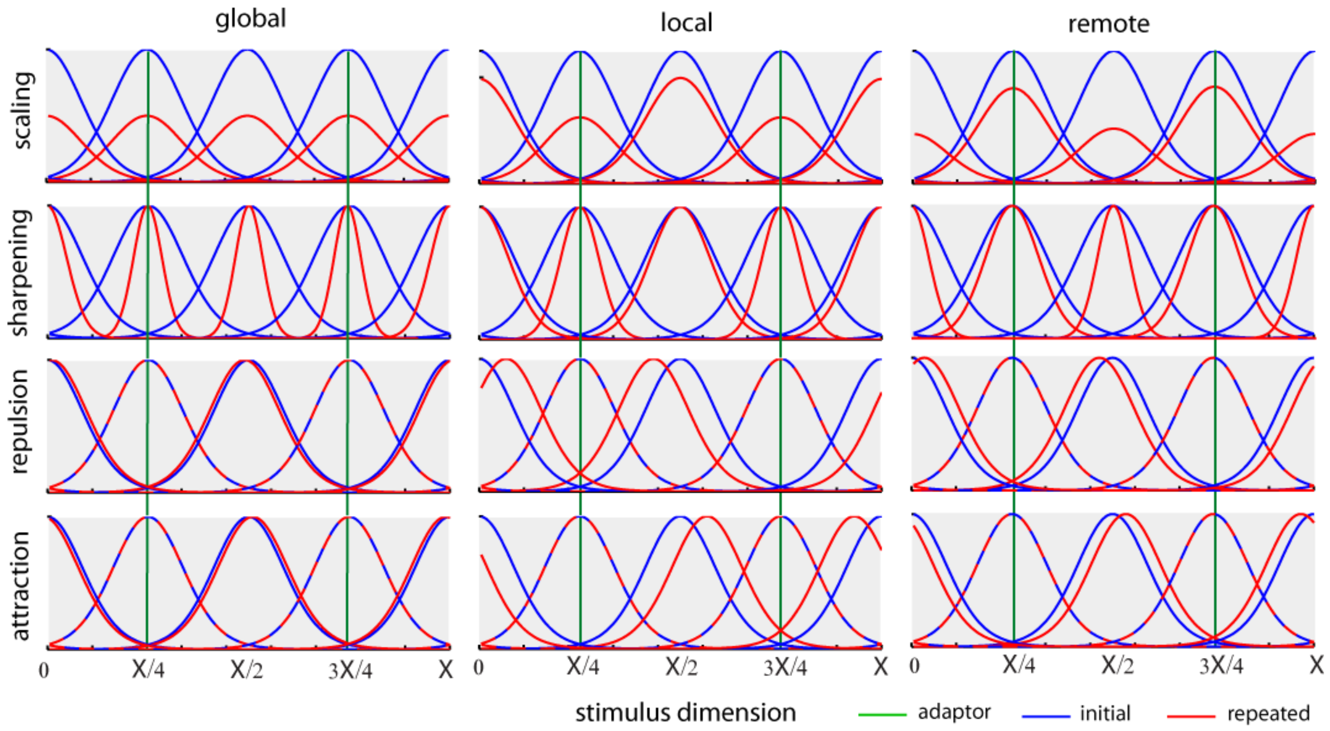

Supplementary Figure 1 - Example tuning curves before (blue) and after (red) after adaptation to both orientations in Experiment 2 according to the twelve different neural models of adaptation. Due to the circular nature of the stimulus dimension orientation, tuning curves were modelled with a von Mises distribution. For illustrative purposes, we only show four neural populations equally-spaced along the stimulus dimension. Mechanisms: scaling - adaptation reduces response amplitude, sharpening - adaptation tightens tuning-curves, repulsion - the peak of tuning-curves moves away from the adapting stimulus, attraction - the peak moves towards the adapting stimulus. Domains: global - all tuning-curves in a voxel are affected, local - tuning-curves close to the adapting stimulus are affected most, remote - tuning-curves close to the adapting stimulus are affected least.

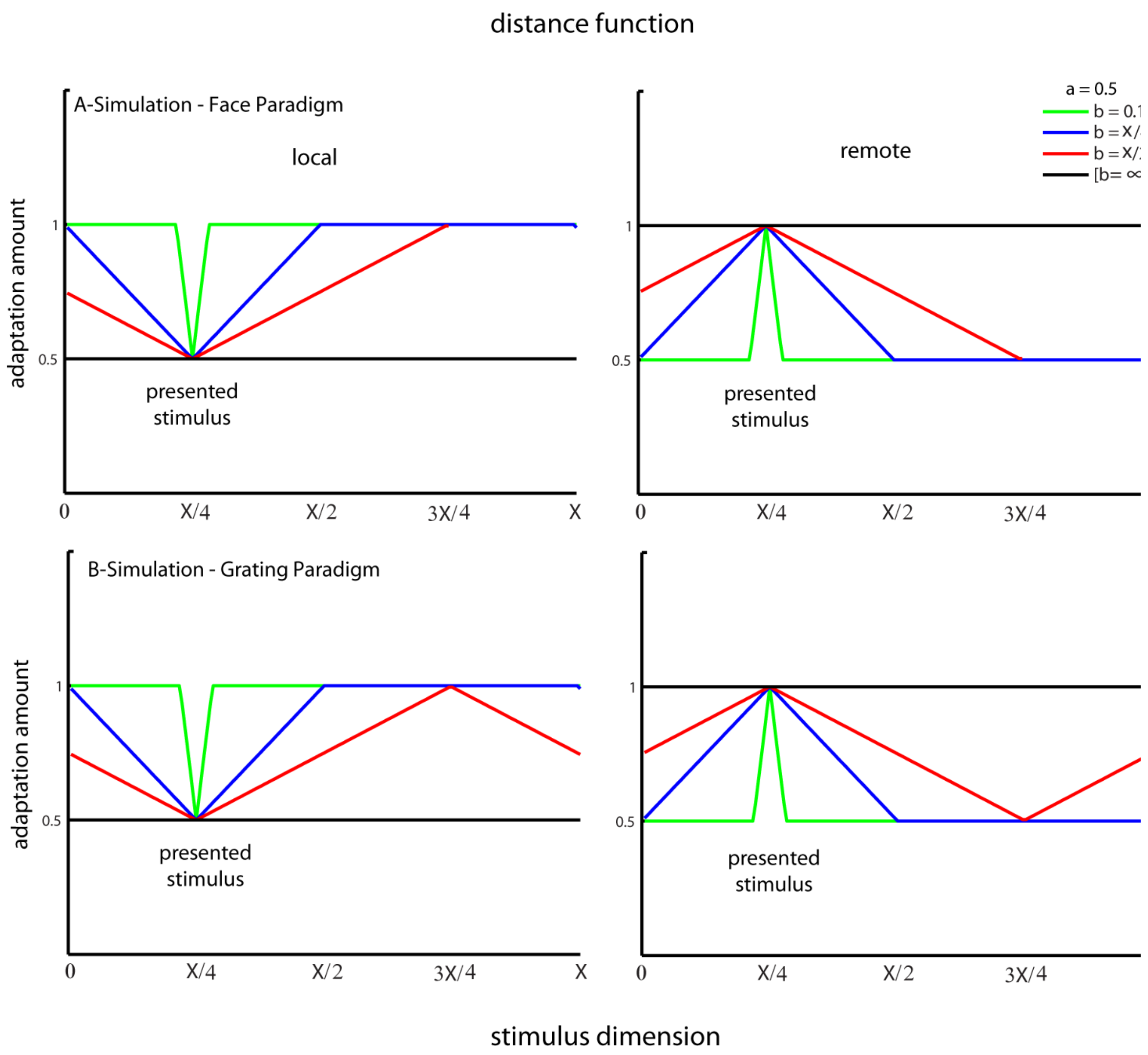

Supplementary Figure 2 - Distance functions, showing how amount of repetition suppression depends on distance between stimulus orientation (x-axis) and neural preference (here X/4) for a non-circular (top) and circular (bottom) dimension for local (left) and remote (right) domains. The *a* parameter is fixed to 0.5, while the *b* parameter is shown from 0.1 to ∞, though note that in our simulations, *b* only ranged from 0.1 to d=X/2.

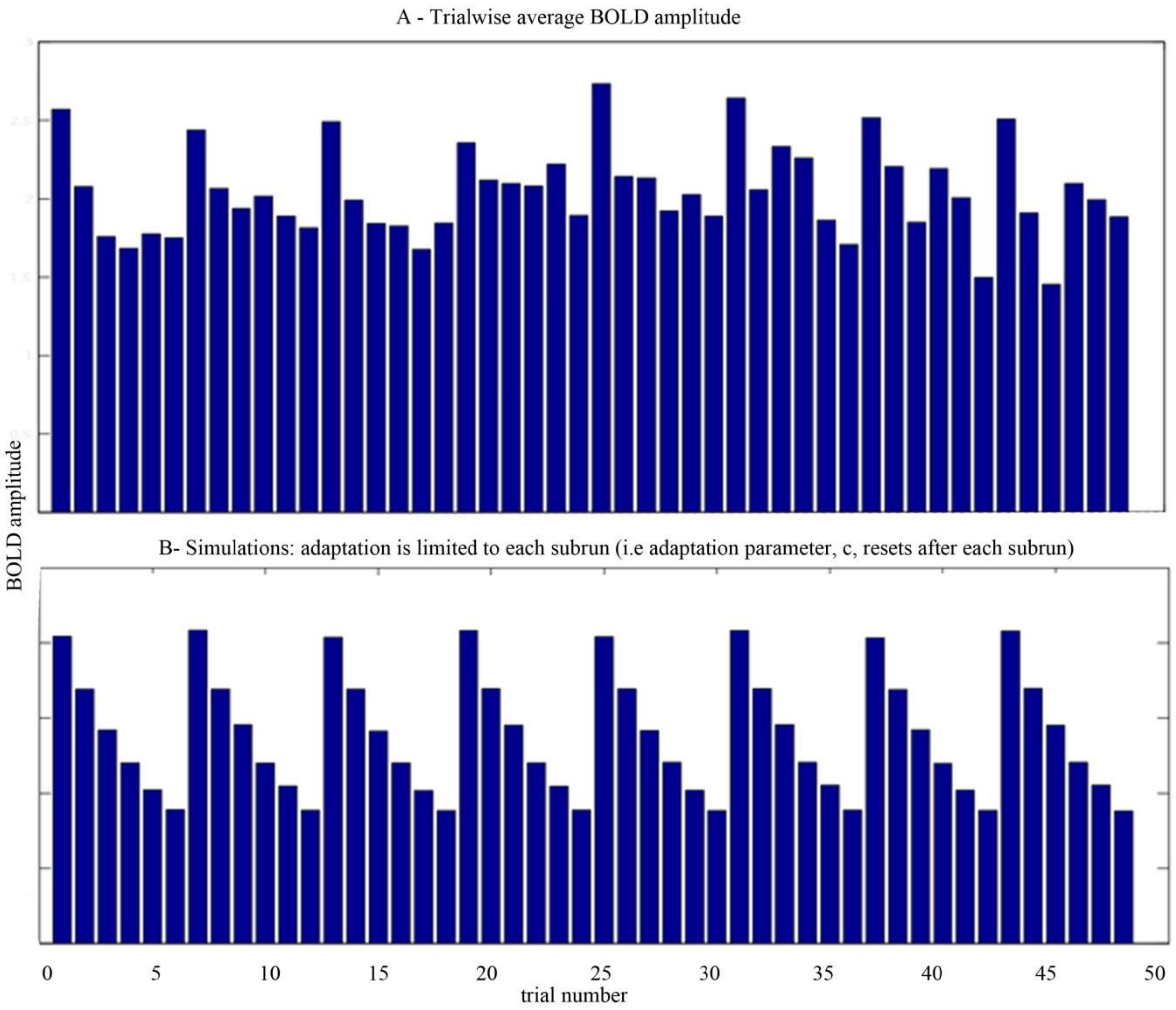

Supplementary Figure 3 - Showing A) adaptation effects were limited to the subruns and the BOLD activity at the start of each subrun was the same as the first trial in each subrun and B) showing the effect of re-setting the adaptation factor between the independent runs in our simulation to match the empirical data.

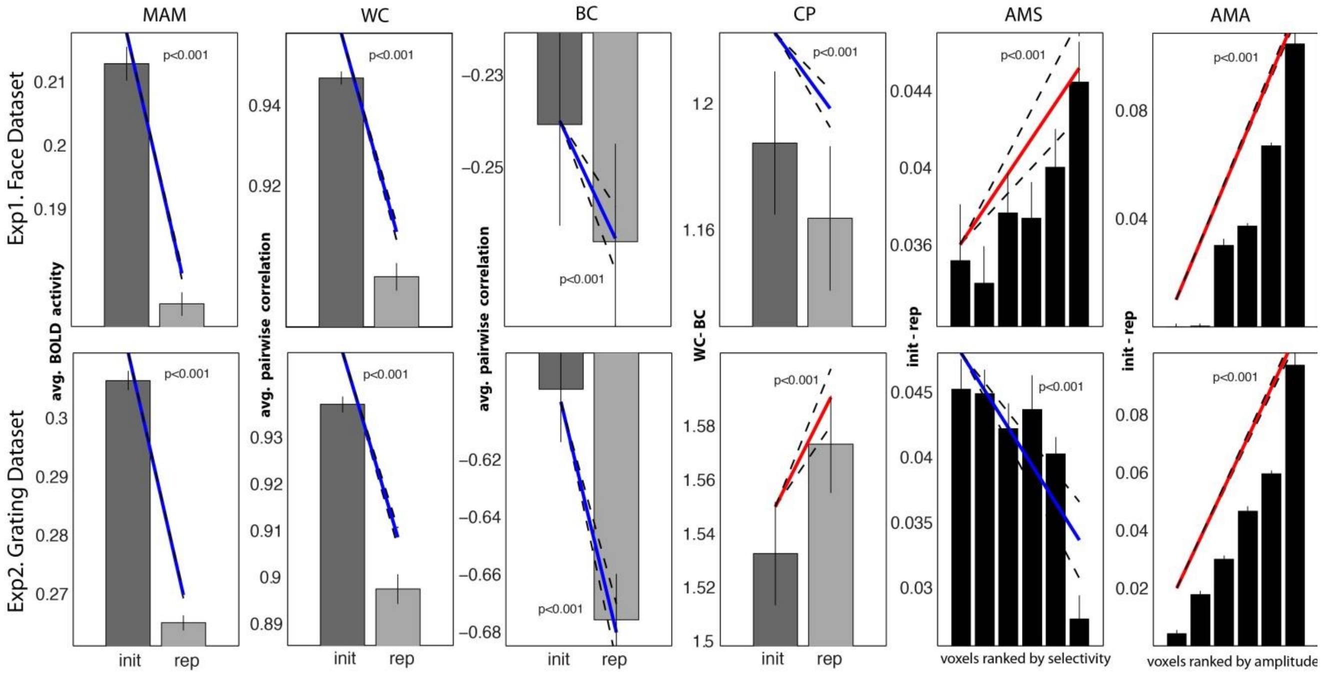

Supplementary Figure 4 - Local scaling prediction for all the 6 criteria with a sample wining parameter: c=0.8, b=0.4, sig=0.4 (grating dataset) and c=0.7, b=0.2, sig=0.2 (Face dataset). Data features: Mean Amplitude Modulation (MAM), Within-class Correlation (WC), between-class correlation (BC), Classification Performance (CP), Amplitude Modulation by Selectivity (AMS) and Amplitude Modulation by Amplitude (AMA). Error bars reflect the 95% confidence interval given between-participant variability. Diagonal lines represent the slope of linear contrasts across conditions (red = positive; blue = negative), with dashed error margins reflecting 95% confidence interval of that slope (equivalent to pairwise difference when only two conditions).

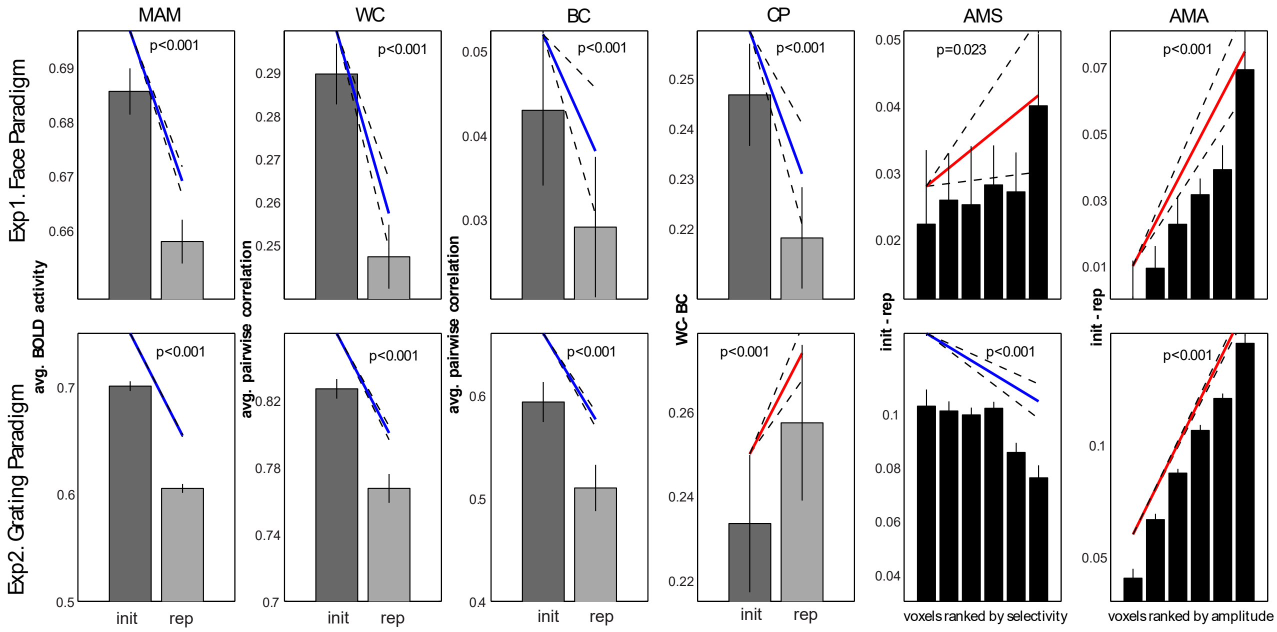

Supplementary Figure 5 - Local scaling prediction for all the 6 criteria after adding correlated neural activity to make BC positive. Around 10% of correlated neural activities (flat curve neurons) were added to the voxels in the face Paradigm simulation, and around 50% added to the grating Paradigm simulation. See the supplementary table 1 for all the wining parameter combinations that achieve a qualitatively similar result to the data. Data features: Mean Amplitude Modulation (MAM), Within-class Correlation (WC), between-class correlation (BC), Classification Performance (CP), Amplitude Modulation by Selectivity (AMS) and Amplitude Modulation by Amplitude (AMA). Error bars reflect the 95% confidence interval given between-participant variability. Diagonal lines represent the slope of linear contrasts across conditions (red = positive; blue = negative), with dashed error margins reflecting 95% confidence interval of that slope (equivalent to pairwise difference when only two conditions).

Supplementary Table

grating dataset				face dataset		
t-stat 99% confidence				t-stat 99% confidence		
a	b	sigma		a	b	sigma
0.7	0.1	0.4		0.6	0.1	0.2
0.8	0.1	0.4		0.7	0.1	0.2
0.7	0.2	0.4		0.8	0.1	0.2
0.8	0.2	0.4		0.9	0.1	0.2
0.8	0.4	0.4		0.6	0.2	0.2
0.8	0.6	0.4		0.7	0.2	0.2
0.8	0.1	0.6		0.8	0.2	0.2
0.9	0.1	0.6		0.9	0.2	0.2
0.8	0.2	0.6		0.6	0.4	0.2
0.9	0.2	0.6		0.7	0.4	0.2
0.8	0.4	0.6		0.8	0.4	0.2
0.9	0.4	0.6		0.9	0.4	0.2
0.8	0.6	0.6		0.6	0.6	0.2
0.9	0.6	0.6		0.7	0.6	0.2
0.9	0.8	0.6		0.8	0.6	0.2
0.9	0.1	0.8		0.9	0.6	0.2
0.9	0.2	0.8		0.6	0.8	0.2
0.9	0.4	0.8		0.7	0.8	0.2
0.9	0.6	0.8		0.8	0.8	0.2
0.9	0.8	0.8		0.9	0.8	0.2
0.9	0.1	1		0.7	1	0.2
0.9	0.2	1		0.7	1.3	0.2
0.9	0.4	1		0.9	1.3	0.4
0.9	0.6	1		0.9	1.7	0.4
0.9	0.8	1				

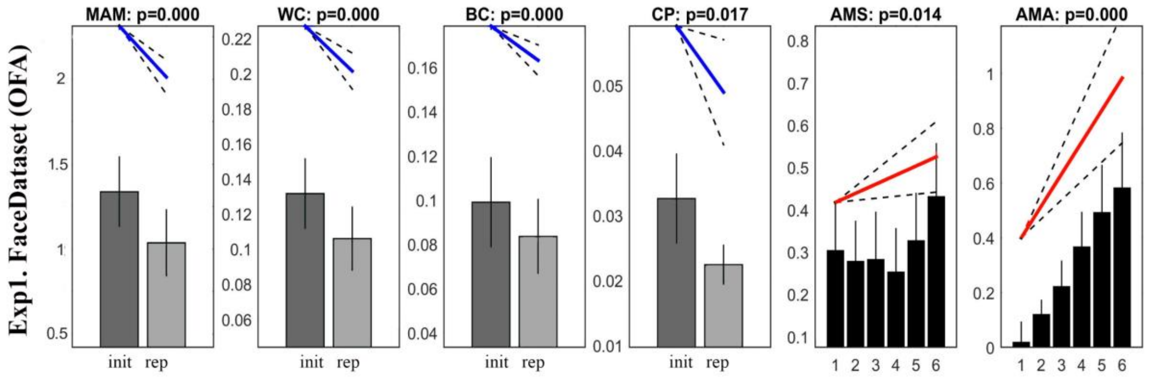

Supplementary Figure 6 - The six data features (columns) for the Face Dataset shown for the occipital face area face-responsive region (OFR) and while comparing initial responses to responses to delayed repetitions. Selecting a different face selective region of interest than FFR does not qualitatively change the effect of repetition on the six data features (for comparison, see Figure 3). Data features: Mean Amplitude Modulation (MAM), Within-class Correlation (WC), between-class correlation (BC), Classification Performance (CP), Amplitude Modulation by Selectivity (AMS) and Amplitude Modulation by Amplitude (AMA). Error bars reflect the 95% confidence interval given between-participant variability. Diagonal lines represent the slope of linear contrasts across conditions (red = positive; blue = negative), with dashed error margins reflecting 95% confidence interval of that slope (equivalent to pairwise difference when only two conditions).

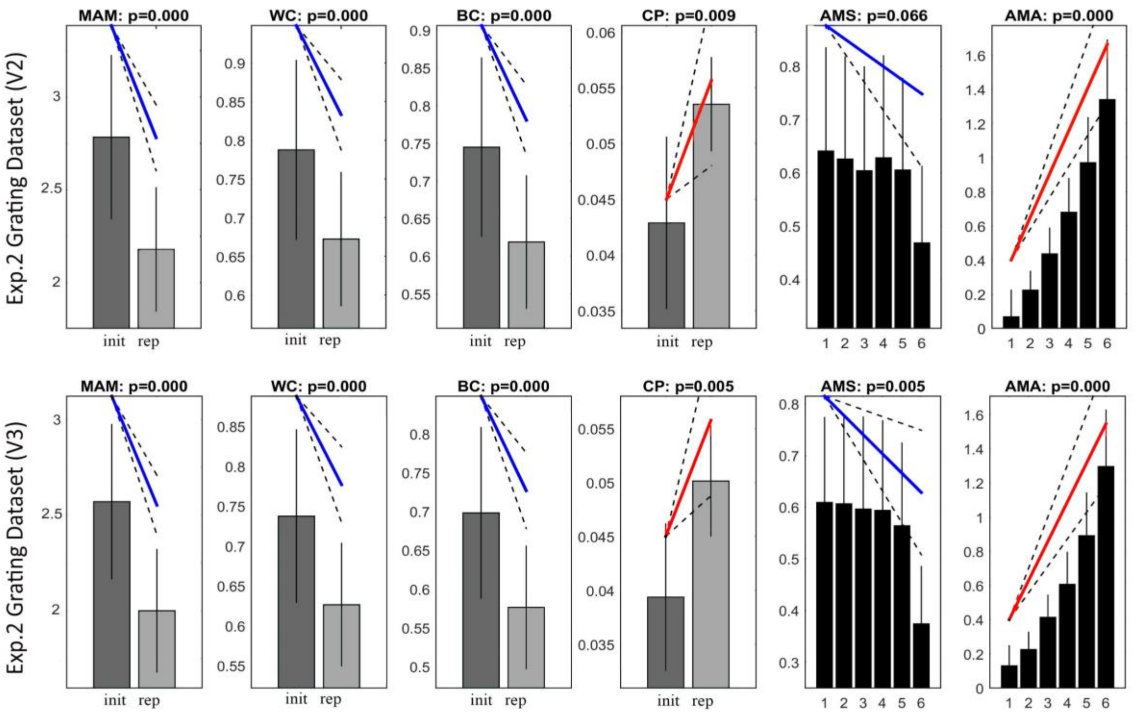

Supplementary Figure 7 - The six data features (columns) for the Grating Dataset shown for visual areas V2 (A) and V3 (B). Selecting different early visual regions of interest than V1 does not qualitatively change the effect of repetition on the six data features (for comparison, see Figure 3). Data features: Mean Amplitude Modulation (MAM), Within-class Correlation (WC), between-class correlation (BC), Classification Performance (CP), Amplitude Modulation by Selectivity (AMS) and Amplitude Modulation by Amplitude (AMA). Error bars reflect the 95% confidence interval given between-participant variability. Diagonal lines represent the slope of linear contrasts across conditions (red = positive; blue = negative), with dashed error margins reflecting 95% confidence interval of that slope (equivalent to pairwise difference when only two conditions).

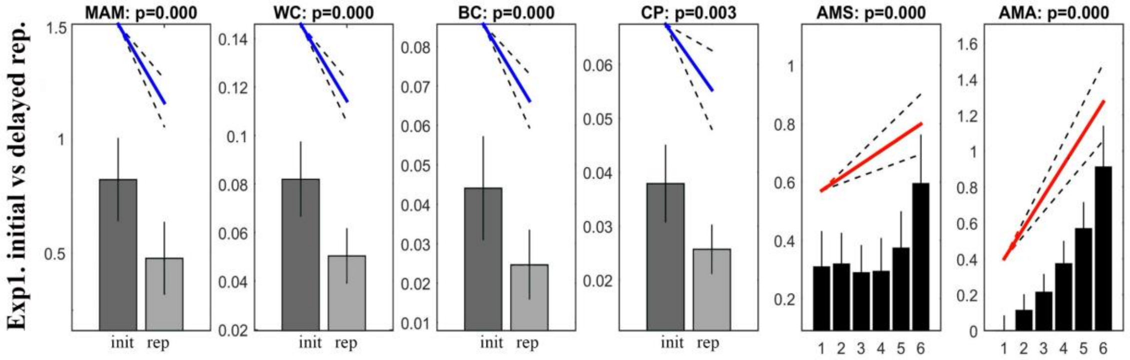

Supplementary Figure 8 - The six data features (columns) for the Face Dataset shown for the fusiform face-responsive region (FFR) while comparing initial responses to responses to delayed repetitions. Comparing initial responses to delayed responses (as opposed to immediate responses) does not qualitatively change the effect of repetition on the six data features (for comparison, see Figure 3). Data features: Mean Amplitude Modulation (MAM), Within-class Correlation (WC), between-class correlation (BC), Classification Performance (CP), Amplitude Modulation by Selectivity (AMS) and Amplitude Modulation by Amplitude (AMA). Error bars reflect the 95% confidence interval given between-participant variability. Diagonal lines represent the slope of linear contrasts across conditions (red = positive; blue = negative), with dashed error margins reflecting 95% confidence interval of that slope (equivalent to pairwise difference when only two conditions).

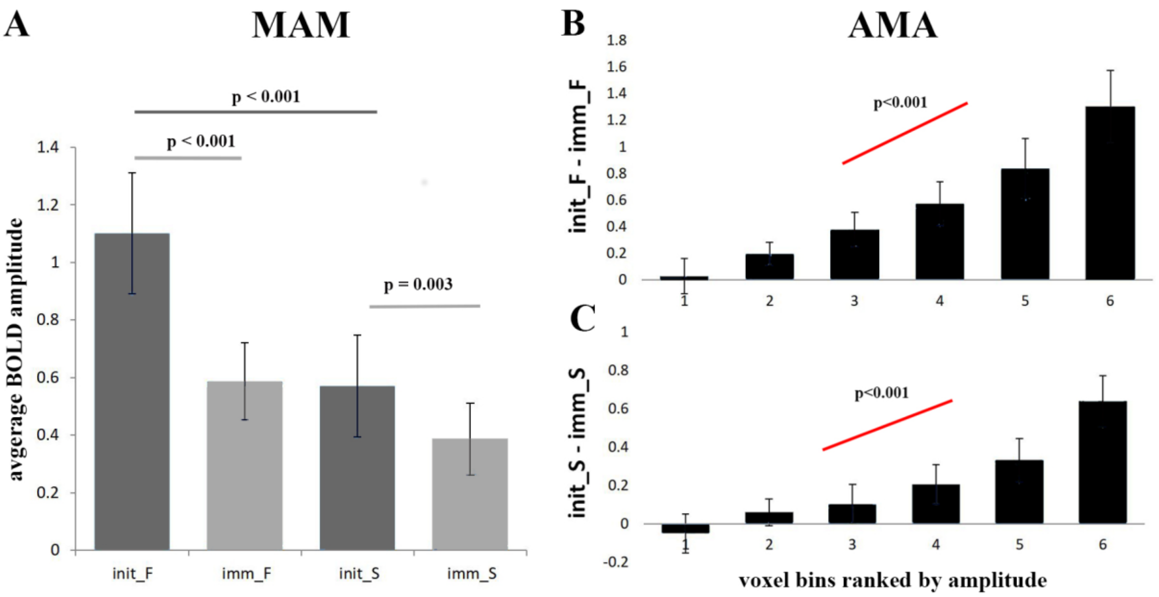

Supplementary Figure - 9 Mean Amplitude Modulation (MAM, A) and Amplitude Modulation by Amplitude (AMA, B) features are shown separately for face (_F) and scrambled face (_S) stimuli based on FFR responses to initial (init) and immediately repeated (imm) stimuli. Neither data feature appears to be qualitatively affected by stimulus type. Data features: Mean Amplitude Modulation (MAM) and Amplitude Modulation by Amplitude (AMA). Error bars reflect the 95% confidence interval given between-participant variability. Diagonal lines represent the slope of linear contrasts across conditions (red = positive; blue = negative).
